# Current state of knowledge on the prevalence of neurodevelopmental disorders in childhood according to the DSM-5: a systematic review in accordance with the PRISMA criteria

**DOI:** 10.1186/s13034-022-00462-1

**Published:** 2022-03-31

**Authors:** Lorena Francés, Javier Quintero, Alberto Fernández, Antoni Ruiz, Jessica Caules, Gabriella Fillon, Amaia Hervás, C. Virgínia Soler

**Affiliations:** 1 Child and Adolescent Psychiatrist, Menorca (Balearic Islands, Spain). Av. Del Metge Camps 20, 07740 Es Mercadal, Balearic Islands Spain; 2 Head of the Psychiatry Service, Infanta Leonor Hospital Madrid, Madrid, Spain; 3 Department of the Complutense, University of Madrid, Madrid, Spain; 4 Department of Legal Medicine, Psychiatry and Pathology, Complutense University of Madrid, Madrid, Spain; 5 University of Barcelona, Barcelona, Spain; 6 Psychopedagogical Center Arrels, Ciutadella, Balearic Islands Spain; 7 Somerset Foundation Trust–National Health System (NHS), London, UK; 8 Child–Adolescent Mental Health Unit at the Mutua Terrasa University Hospital, Catalonia, Spain; 9 Saint George Hospital in London, London, UK; 10 Child-Adolescent Psychiatry at Maudsley Hospital, London, UK; 11 Dalt Sant Joan Center (Mahón), Illes Balears, Spain

**Keywords:** Neurodevelopmental disorders, Prevalence, Childhood, Diagnosis, Autism spectrum disorder, ADHD, Learning disability, Language disorder, Motor disorders, Intellectual disability

## Abstract

**Objective:**

To interpret the current evidence on the prevalence of neurodevelopmental disorders (NDDs) through a systematic review based on both DSM-5 (2013) and PRISMA criteria.

**Method:**

Empirical studies complying with the PRISMA guidelines were identified from four databases (PubMed, Scopus, Science Direct, and ProQuest) and systematically reviewed. In total, 17 articles were selected for the study.

**Results:**

In the scientific literature, there have been only a few studies measuring the prevalence of NDDs according to the DSM-5 (2013) criteria in people under 18 years old. The reported prevalence rates were as follows: intellectual disability (ID), 0.63%; attention-deficit/hyperactivity disorder (ADHD), 5–11%; autism spectrum disorder (ASD), 0.70–3%; specific learning disorder (SLD), 3–10%; communication disorders (CDs), 1–3.42%; and motor disorders (MDs), 0.76–17%. Although there is extensive literature on specific disorders, NDDs have rarely been assessed as a whole.

All of the reviewed studies support the idea that such disorders can be considered chronic, heterogeneous, underdiagnosed conditions and that comorbidity of multiple NDDs is the norm. Likewise, it is estimated that the prevalence of the most studied disorders, such as ADHD, ASD and SLD, remains stable over time and is consistent in different cultures, ages, ethnicities and sexes.

**Conclusion:**

The studies reviewed lead us to conclude that the prevalence rate of NDDs fluctuates globally between 4.70 and 88.50%; these variations depend on methodological aspects such as estimation procedures, as well as on sociocontextual phenomena. It is also important to consider that the prevalence found is probably highly influenced by the activity of the countries in the diagnosis and training of professionals who care for children and adolescents. Hence, there is a need for a secondary intervention in the fields of public health and education to minimize socioemotional consequences, prevent academic failure, and reduce the economic cost to society.

**Supplementary Information:**

The online version contains supplementary material available at 10.1186/s13034-022-00462-1.

## Background

The Diagnostic and Statistical Manual of Mental Disorders, 5th Edition (DSM-5; American Psychiatric Association, 2013) introduced a new diagnostic category called neurodevelopmental disorders (NDDs), a group of disorders that commonly begin in childhood and can be chronic conditions that persist for life.

This new approach is committed to the inclusion of NDDs within a heterogeneous and dimensional group, leaving behind the categorical classifications of the DSM 4^th^ Edition Text Revision (DSM-IV-TR; American Psychiatric Association, 2004) and the International Statistical Classification of Diseases and Related Health Problems (ICD-10; World Health Organization, 1992). It is expected that the next ICD edition (ICD-11) will unify its criteria with those of the DSM-5 (2013). Finally, a revised DSM-5 (i.e. DSM-5-TR) will also be published in 2022.

As mentioned above, the category of NDDs includes disorders that manifest in a general way in almost all developmental domains, such as intellectual disability (ID), as well as those that affect more specific domains, such as attention-deficit/hyperactivity disorder (ADHD), autistic spectrum disorder (ASD), communication disorders (CD), specific learning disorder (SLD, including difficulties in reading, writing and mathematics), and motor disorders (MDs, such as Tics, Tourette's and stereotypic disorders), among others.

The current detection rates of developmental disorders are lower than their real prevalence, according to Zwaigenbaum and Penner [1]. A study by Petersen et al., 2014, noted that these disorders affect 15–20% of the child population, which is why they constitute a common reason for consultation in childhood and adolescence.

In the United States, according to data published by the National Center for Health Statistics (NCHS) in 2015, an estimated 15% of children aged 3 to 17 years are affected by NDDs.

In previous studies, the prevalence rates of the most common NDDs were estimated as follows: ADHD = 7.9–9.5% [3, 4]; ASD = 0.7–2.2% [4–6]; SLD (or developmental dyslexia [DD]) = 1.2–24% [7, 8]; and motor coordination disorder = 1.4–19% [9, 10]. Furthermore, the prevalence rates reported for various disorders within the same study did not include the rates of coexistence between disorders [11]. Likewise, there is disparity and diversity in the methods used by the scientific community to estimate prevalence. To determine the prevalence of these disorders, surveys have been applied to different populations (general, clinical, school), and different professionals have performed the assessments (medical specialists, teachers, school counsellors); very few studies have assessed and directly examined the individuals, with most studies merely extrapolating conclusions from specific clinical and/or population databases. In this way, studies reach conclusions that may reflect certain inherent biases. Therefore, according to Thomas R. et al. [2, 3, 11], systematic reviews would be one of the best solutions to this problem.

Clinical experience leads us to believe that it is rare for a single NDD to occur in isolation; rather, there is overlap between different disorders (homotypic comorbidity) and with other psychiatric psychopathologies (heterotypic comorbidity). The study of NDDs as a whole and in the context of their comorbidities is necessary to approximate clinical reality and to estimate the true scope of each specific disorder. Finally, it is possible that various target disorders are initially masked in some patients but become clinically apparent with age [12, 13].

## Patients and methods

This work is a review of the published scientific literature on paediatrics, child and adolescent psychiatry and all journals related to *NDDs,* specifically in relation to the epidemiology of NDDs as defined by the DSM-5 (2013).

**Fig. 1:**
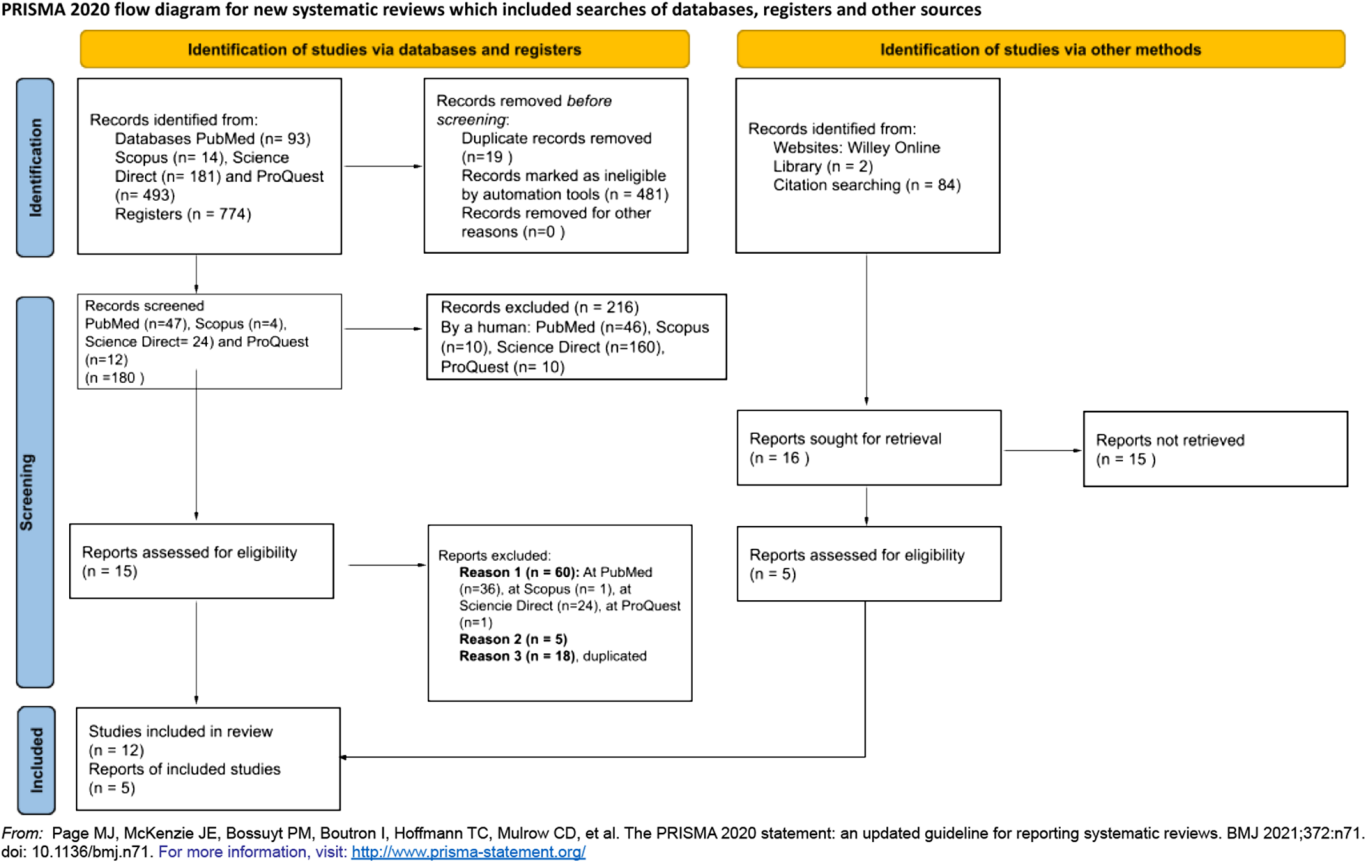
Flow chart

The review follows the guidelines of the Preferred Reporting Items for Systematic Reviews and Meta-Analyses (PRISMA) declaration for the correct performance of systematic reviews [14] PRISMA are considered a formal research process that ensures replicability in the results. It aims to provide a solid and universal protocol for systematic review and documents reviews transparently. See Fig. 1, Flow Chart.

The selection process identified 17 articles that were deemed appropriate. These articles report on aspects of the prevalence of NDDs worldwide, spanning Asia, Europe, Australia, the USA, Latin America and Africa. Such breadth is important, considering the possible effects of socioeconomic resources on the diagnosis and development of certain conditions.

Methodologically, the studies collected their information from a variety of sources: surveys (of parents and/or teachers), diagnostic records in health systems, records from special schools, and records of prescribed pharmacological treatments provided by public health systems and private insurance (USA). However, the possible biases of our selection methods must be considered, since, in the included meta-analyses, different meta-analytical techniques are used to estimate and unify prevalence rates by group and homogenize the samples, which vary depending on geographical areas, sex, ethnicity, and population type.

The data in the included studies were collected according to various diagnostic criteria; the most widely used are the DSM-IV-TR and ICD-10 manuals. The change in criteria with the publication of the DSM-5 (2013) compels us to consider the possibility that the literature reflects a persistent underdiagnosis of comorbidities (Additional file 1).

## Results^1^

The analysis that we will present below is arranged in the order that we consider the most appropriate to facilitate an understanding of the subject, and we have attempted to integrate and distil the results into simple and understandable points. The main results are shown in Summary Table 1.

**Table 1:** Summary table

AuthorYear of publication	Geographical area	Sample	NDDs considered	Sample age	Time window	Differences in sex	Methodology/ type of study	Diagnostic criteria	Sources of information	Type of population
Bosch et al., 2021	Catalonia (Spain): 28 schools	6834 students	All NDDs according to DSM-5: ID, ASD, ADHD, SLD, CDs and MDs	5–17 years	Not specified; two-phase study, initiated in 2011	Yes, ASD and MD were more common in boys than in girls	Prevalence study	DSM-5	Directly from the child through the administration of specific tests in phase 1; assessment by expert psychiatrists and neuropsychologists in phase 2	School: public and private. Rural and urban
Bita et al., 2018	LAMIC: Africa n = 16 (31.4%) (77.6%), Asia–Pacific n = 19 (37.3%), Western Europe n = 7 (13.7%), Latin America n = 7 (13.7%), multisite n = 2 (3.9%)	274,028 subjects 51 studies	ADHD ASD Other neurological conditions: epilepsy, hearing impairment, visual impairment, ADHD, behavioural/emotional problems, mental disorders	< 19 years	Since 1995	Not estimated	Systematic review and meta-analysis	None	Multiple surveys	General (Rural and urban)
Arora et al., 2018	India (5 regions): north-central (Palwal), north (Kangra), east (Dhenkanal), west (north Goa) and south (Hyderabad)	3964 children (83.9% of all invited candidates; 99.4% of all enrolled subjects) Composition: 2,006 boys and 1,958 girls	NDD: visual impairment, epilepsy, neuromotor impairment including cerebral palsy, hearing impairment, speech and language disorders, ASD and ID Children from 6 to 9 years old: ADHD and learning disorders	2–9 years	Data were collected between 5 December 2011 and 27 September 2012	No significant difference Prevalence: 12.4% (95% CI 10.2%-15.0%) in boys versus 10.2% (95% CI 8.4%-12.2%) in girls (p = 0.146)	Prevalence study	DSM-IV-TR Validated tools for ASD, ADHD and epilepsy (INCLEN Diagnostic Tool)	Cross-sectional survey of children's parents and interviews by accredited professionals, demographic details extracted in the 2011 Indian census	General (rural and urban)
Carballal et al., 2017	Galicia (Spain)	1286 children	Child and adolescent psychiatric pathology	0–14 years	Between September and November 2015	Not determined	Observational, descriptive and cross-sectional study	DSM-IV-TR	Interview and review of clinical history according to DSM-IV-TR axes Professional evaluators: 57% school counsellors, 42% child–adolescent mental health unit, 37% public neuropaediatricians, 33.6% schoolteachers, 27.4% speech therapists and 15% early care services	Patients receiving primary mental health services and follow-up by child–adolescent mental health unit
Wang et al., 2017	China: East China (20 studies), Central China (10), South China [11], Southwest China (seven), North China (six), Northwest China (five), Northeast China (four) and Hong Kong/Taiwan (four)	275,502 subjects out of 334,000 recruited 67 studies	ADHD	Up to 18 years	30 years	Not determined	Systematic review and meta-analysis	DSM (n = 86.57%) DSM-III, DSM-III-R, DSM-IV and DSM-5 CCMB-2, CCMB-3, ICD-9	Clinical interviews were administered in 58.2% (n = 39) of the studies analysed Medical information was collected from the children (n = 4), teachers and parents in the remaining studies	General
Catalan-Lópet al., 2012	Spain	13,026 subjects 14 studies	ADHD	< 18 years	Original studies published between January 1980 and August 2011	Male:female ratio of 4:1 in four studies and 2:1 in three studies Higher prevalence in males	Systematic review and meta-analysis	DSM-III-R, DSM-IV and ICD-10	Symptom-based questionnaires and scales Parents and teachers In half of the studies, data collection was divided into 2 stages: (1) psychometric screening and (2) clinical confirmation using standardized diagnostic criteria	General (30%) and school
Pérez Crespo et al., 2019	Catalonia (Spain)	1,326,666 children (51.5% boys and 48.5% girls)	ASD	2–17 years The most common age range was 6–10 years (48.2%), followed by 2–5 years (30.3%) and 11–17 years (21.5%)	Between 2009 and 2017	4.5 times more common in boys (12,647 boys versus 2,819 girls)	Retrospective analytical cohort study	ICD-9 ICD-9 codes 299.0, 299.1, 299.8, and 299.9	Based on ICD diagnoses in the Catalan Health System	Clinic patients
Kita et al., 2020	Japan	3852 children	NDD: ADHD, ASD, SLD (DD) and coordination disorder First study to measure comorbidity between them The prevalence of ODD was also estimated because of its high comorbidity with ADHD	6–9 years	2015 (cross-sectional)	Not calculated	Cross-sectional prevalence study (2015) conducted in schools through parents and teachers, with response rates of 63.9% and 22.5%, respectively	DSM-5 SNAP-IV for ADHD SRS-2 for ASD RWC scale for dyslexia (SLD) Movement Assessment Battery for Kids – Second Edition Checklist (MC) for Motor Disorders	Surveys Based on parent–teacher rating scale questionnaires Two evaluators All rates of agreement on children with suspected NDDs were low (range, 6–16%)	Pupil, community
Fleming et al., 2020	Scotland	766,244 subjects (390,290 [50.9%] boys; 375,954 [49.1%] girls)	ASD ID ADHD Depression	4–19 years	Subjects attended school between 2009 and 2013	Multimorbidity was more common among boys Girls were less likely than boys to have multimorbidity but experienced a greater adverse impact on educational outcomes	Cross-sectional cohort study	ASD from additional support needs ADHD if they have received treatment with stimulants or nonstimulants Depression if they have received antidepressant treatment	Educational and health databases (Scottish Educational Data Exchange Unit **(**ScotXed**)** and 2 health databases through ISD (Information Services Division)	Pupil
Hansen et al., 2018	Norway	407 children	Prevalence rates of NDDs (ADHD, TD, ASD) and comorbid disorders Comorbidity between different NDDs	7–13 years	Between September 2007 and February 2009	Boys constituted a significant majority of referred children (66.3%). There were no significant differences in gender distribution or mean age between the overall NDD group and the psychiatric disorder group without NDDs or between any two specific NDD groups. Among children with ADHD, a significantly higher proportion of girls than boys had comorbid anxiety disorders	Cross-sectional study	DSM-IV	Cross-sectional interviews of parents (at a single timepoint) by experienced doctors Instruments: validated diagnoses in children, Schedule for Affective Disorders and Schizophrenia – Present and Lifetime version (Kiddie-SADS-PL), DSM-IV version	Clinical: Consultation External CAMHS
Dalsgaard et al., 2020	Denmark	14.4 million person-years of follow-up	All mental health disorders	Up to 18 years	From 1 January 1995 to December 31, 2015	Anxiety was the most common diagnosis in girls (7.85%) ADHD was the most common disorder in boys (5.90%). The overall risk of being diagnosed with a mental disorder before 6 years of age was 2.13% overall, with a higher risk in boys (2.79%) than in girls (1.45%)	Cohort study	ICD-10 Classification of Mental and Behavioural disorders: Diagnostic Criteria for Research (ICD-10-DCR), ICD-10	Comprehensive clinical evaluations of all mental disorders by interdisciplinary clinical teams including child and adolescent psychiatrists	Departments in public hospitals Records in the Health System Central Registry of Psychiatric Investigations of Denmark and National Registry of Patients of Denmark
Sayal et al., 2017	Community in general, international studies (USA, UK, Japan, Norway, Ireland, Denmark, Scotland, Sweden, Israel, Netherlands, Germany, Thailand and Australia)	7 systematic reviews	ADHD	Two age groups: children aged ≤ 6 years and adolescents aged up to 18 years transitioning to adult services	Publications between 1996 and 2016	More common in males by a factor of 2–3	Review	DSM-IV	Parent ratings, teacher assessments, or best-estimate diagnostic procedures Data on pharmacological prescriptions	Primary care School Insurance Private practice
Saito et al., 2020	Hirosaki, Japan	5016 children were eligible 3954 children completed and returned the screening package 559 children were assessed comprehensively in person	ASD and its comorbidity with other NDDs	5 years	2013–2016	The raw male:female prevalence ratio was 2.2:1 Common comorbid conditions included ADHD (50.6%, male:female = 2.4:1), DCD (63.2% male:female = 2.1:1), ID (36.8%, male:female = 1.7:1), and borderline intellectual functioning (20.7%, male*:*female = 2.6:1)	Sequential-cross-sectional design study	DSM-5 Autism Spectrum Screening Questionnaire (ASSQ), Strengths and Difficulties Questionnaire (SDQ), ADHD IV Rating Scale (ADHD-RS-IV), Developmental Coordination Disorder Questionnaire (DCDQ), and Parental Stress Index (PSI) DISC and ADOS for ASD WISC-IV MABC-2: For T. of movement	Comprehensive assessment, which included interviews with children and parents, behavioural observation, and tests of cognitive and motor function. All cases were reviewed by a multidisciplinary research team	HFC Facts (Hirosaki Five- year-old Children Developmental Health Check-up)
Shriberg et al., 2019	USA	346 participants	The objective of this research was to use measurements and analyses in a diagnostic classification system to estimate the prevalence of speech and language disorders in convenience samples of speakers with one of the eight types of complex NDD	Average of 13.3 years	30 years	No sex differences were detected in the prevalence of disorders	Prevalence study	SSD (Speech Sound Disorders)	Audio recordings of speech Narrow phonetic transcription, prosody–speech coding, and acoustic analysis Research specialists in the field	Population- specific database of participants recruited for studies of genetic and behavioural disorders of speech sound production (i.e., excluding disfluency)
Murphy et al., 2015	Midwestern states, USA	136 children	Language disability Preschool language and early literacy skills One-quarter of children (21%, n = 29; 1%, n = 2 missing information) had moderate disabilities, including ASD (n = 13), ADHD (n = 2), Down syndrome (n = 2), developmental delay (n = 2), hearing loss (n = 1) and foetal alcohol syndrome (n = 1)	Average of 56 months (SD = 4.5, range 48–69 months)	Cross-sectional	Not determined	Retrospective prevalence study	No DSM; criteria were specified	Experienced professionals and caregivers Word recognition task	Pupil population: children with language disabilities attending special education schools
Fortes et al., 2015	Low- and middle-income areas of Brazil	1618 children and adolescents	Learning disorders and their comorbidity with other homo- and heterotypic psychiatric disorders	at least 9 years of schooling	Cross-sectional	Yes, learning disorder and ADHD were more prevalent in males than in females Significant differences in prevalence rates were detected between cities, and several sociodemographic correlates (age, sex, IQ and socioeconomic status) were significantly associated with SLD with global impairment in this sample	Cross-sectional prevalence study	DSM-5	Direct observation by qualified psychologists	Pupil
Faraone et al., 2021	Worldwide	Studies in the analysis included > 2000 participants	ADHD	All ages	20 years	ADHD is more common in men than in women. The meta-analysis examined parents' ratings of symptoms in 29 studies with more than 42,000 participants, as well as teacher ratings in 24 studies with more than 56,000 participants; a male:female ratio of 2:1 was found in youth	Systematic review, international consensus of ADHD We reviewed studies with more than 2000 participants or meta-analyses of five or more studies or 2000 or more participants	DSM	Studies with scientific evidence	General, clinical, pupil

We will present the global selection of studies by diagnostic themes in the following order:

### Studies regarding NDDs in general:

Seven studies evaluated the global prevalence of NDDs; 6 of these works were prevalence studies, and the remaining one was a systematic review and meta-analysis. In a systematic review and meta-analysis [15], the prevalence of NDDs was estimated in low- and middle-income countries (hereafter, LAMIC); it was concluded that the burden of NDDs in LAMIC is considerable and that there is a lack of reliable epidemiological data on some NDDs, such as ASD, which may lead to underestimation of the true burden of these conditions in LAMIC. Mental disorders such as ADHD and ASD have rarely been reported, and more studies are needed, particularly in Africa and Latin America, to provide reliable estimates, as neurological conditions such as epilepsy generally have more reliable estimates than mental disorders.

In 2021, the research group of Bosch et al. [16] published the first study reporting the prevalence rates of all NDDs in Spain; the rates were determined through direct examinations of 6834 students aged 5–17 years from 28 schools in Catalonia. The study concluded that these conditions were underdiagnosed, and the following prevalence rates were obtained: ID, 0.63%; CD, 1.05%; ASD, 0.70%; ADHD, 9.92%; SLD, 10.0%; and MD, 0.76%.

In India, a study published by Arora et al. [17] in 2018 assessed the prevalence of several NDDs: visual impairment, epilepsy, neuromotor disability (including cerebral palsy), hearing impairment, speech and language disorders, ASD and ID. Additionally, children aged 6 to 9 years were screened for ADHD and learning disorders. The prevalence of NDDs varied between locations. The site-specific prevalence of these seven classes of NDDs in children aged 2 to 6 years ranged from 2.9% to 18.7%, while children aged 6 to 9 years showed a 6.5% to 18.5% prevalence of the nine NDDs. Hearing impairment and ID were the most common NDDs. Approximately one-fifth of children with NDDs suffer from two or more. The pooled estimates for NDDs across all sites for NDDs were 9.2% and 13.6% in children ranging from 2 to 6 and 6 to 9 years, respectively, with no significant differences by gender, rural/urban residence, or religion. Hearing impairment, ID, speech and language disorders, epilepsy, and learning disorders were found to be common NDDs across all sites. Among children with NDDs, 21.7% had two or more; comorbid NDDs were most common in children with ASD (79.6%), cerebral palsy (74.2%), ID (56.9%) and epilepsy (55.1%).

In Japan, Kita et al. [11] conducted the first study measuring comorbidity among ADHD, ASD, SLD (DD) and CD. Oppositional defiant disorder (ODD) was also evaluated due to its high comorbidity with ADHD. The results indicated that 0.4% of children had comorbid ADHD, ASD and SLD. The prevalence rates of ADHD ranged from 6.3% to 6.5% depending on the rating methods. The parent-reported ASD prevalence rate was approximately 1.9%.

The comorbidity rates between ADHD and other disorders were 1.1% for ASD and 0.6% for dyslexia or SLD with reading and writing difficulties, according to parent-completed rating scales. These rates were not significantly different from those based on teacher rating scales: 2.1% (ADHD × ASD) and 1.2% (ADHD × dyslexia; P = 0.09 and 0.23, respectively). Regarding triple comorbidity, the parents reported that 0.2% of the children had concurrent ADHD, ASD, and dyslexia, which was lower than the rate evaluated by their teachers (P < 0.001).

In Scotland, Fleming et al. [18] estimated the prevalence rates of ASD, ID, ADHD and depression. The results indicated neurodevelopmental comorbidity (2 of these conditions) in 0.6% of the children, with ASD and ID being the most common combination.

A total of 4.7% had at least one of the interest conditions, and 0.6% had 2 or more conditions. Of the children who had ASD, 33.0% had at least one other condition. Of the children with ADHD, 29.2% had at least one comorbidity. Of the children with ID, 16.5% had comorbidities, and of the children with depression, 10.6% had comorbidities.

The most common combination was ASD with ID, which occurred in 0.3% of children; 81.0% of the children with this combination were boys. Multimorbidity was the most common form of coexisting ASD and ID. ADHD, by itself or coexisting with other conditions, was the factor with the greatest weight in increasing exclusion from school. Multimorbidity was more common among men, with the prevalence increasing with deprivation. Girls were less likely to have multimorbidity, although with a greater negative impact on educational outcomes compared to boys.

In Norway, Hansen et al. [19] estimated the prevalence rates of NDDs (ADHD, tic disorder (TD), ASD, and homotypic and heterotypic comorbid disorders). Children with NDDs constituted 55.5% of children referred to Child and Adolescent Mental Health Services (CAMHS).

Prevalence estimates for ADHD ranged from 20.8% to 44.5%, TD from 1.8% to 17.7%, and ASD from 2.3 to 10.3%. Despite the different diagnostic procedures between studies, ADHD clearly appears to be the most frequent NDD found. One or more NDDs were diagnosed in 55.5%, of whom 69.9% were boys; ADHD in 44.5%, of whom 68.5% were boys; TD in 17.7%, of whom 77.8% were boys; and ASD in 6.1%, of whom 76% were boys. Among children with NDDs, 31.0% had only one NDD without a comorbid disorder, 21.7% had more than one NDD, and 58% had a comorbid non-NDD psychiatric disorder. Males constituted a significant majority of referred children (66.3%).

In Spain, Carballal et al. [20] studied the prevalence of infants through adolescent psychiatric pathology in primary care consultations with follow-up by infant-juvenile mental health units. They found that the most frequent pathologies were ADHD (5.36%), language disorders (3.42%), learning disorders (3.26%), anxiety and depressive disorders (2.4%) and conduct disorders (1.87%). Forty-seven percent had comorbidities with another mental disorder; most of these children required multiprofessional care in the social, health and educational fields, and 33% received psychopharmacological treatment.

### Studies regarding ADHD:

ADHD continues to be the most studied NDD; accordingly, this study was able to locate 4 systematic reviews and meta-analyses on the topic. The most relevant findings are summarized in the attached summary table. In China (Wang et al., 2017) [21], the overall combined prevalence of ADHD among children and adolescents was 6.26%. In Spain, the overall combined prevalence of ADHD was estimated at 6.8% [22, 23] estimated that the global prevalence of ADHD is 5%, with a peak at 9 years, and suggested that the range reported in the community prevalence of ADHD (2.2–7.2%) reflects the variation in the study methodology.

The World Federation of ADHD International Consensus Statement study [24] found that 5.9% of young people meet the diagnostic criteria for ADHD. That study did not find an increase in the prevalence of ADHD in children and adolescents over the past three decades. In black youth under 18 years of age, an ADHD prevalence of 14% was obtained. Additionally, ADHD was more common in male youth than in female youth (2:1).

### Studies regarding ASD:

Our review included 2 articles on the topic of ASD. A recent study carried out in Catalonia [25] revealed an overall ASD prevalence of 1.23% in 2017, with 1.95% for boys and 0.46% for girls. The highest prevalence (1.80%) was found in children from 11 to 17 years old. Overall, the prevalence of ASD observed in that study was 1.23%, with a male:female ratio of 4.5:1, which is consistent with previous studies. Saito et al. [26] carried out a study assessing ASD and comorbid NDDs in 5-year-old children in Japan according to the DSM-5 (2013); they determined that the adjusted prevalence of ASD was 3.22%. Only 11.5% of children with ASD were free of comorbid NDDs; the remaining 88.5% had at least one other NDD (that is, ADHD, developmental coordination *disorder* (DCD), ID, and/or borderline intellectual functioning). Notably, 23% of children with ASD also had two other NDDs concurrently.

### Studies regarding the prevalence of child and adolescent psychopathology:

Carballal et al. [20] and Dalsgaard et al. [27] examined the prevalence of child and adolescent psychopathology; these studies are commented on in the summary table.

### Other topics of interest regarding diagnostic approaches:

See the summary table for comments on the work of Shriberg et al. [28], Fortes S. et al. [29] and Murphy et al. [30].

## Conclusions

The objective of this systematic review was to determine the prevalence of NDDs to estimate their global prevalence. Few studies have considered the DSM-5 classification (APA, 2013); our review found only 2 such studies [16, 26].

The criteria used by the different publications varied greatly, and the processes used to measure the indicators were often not made explicit. There has been little direct assessment and diagnostic certainty in the clinical population. Furthermore, studies usually did not take into account the complexity and comorbidities of the disorders studied; instead, disorders tended to be analysed individually. Secondary sources are important as complementary resources for diagnosis, and prevalence studies with direct sources are lacking. This review identified only five studies that clearly calculated the prevalence of NDDs through direct examinations of the studied population [16, 26, 29] and covered the most prevalent disorders within the NDD group according to the DSM-5 (2013). Two other studies [28, 30] examined the populations directly but did not follow the DSM-5 (2013) criteria and included smaller samples. In the other studies chosen, the prevalence tended to be established by indirect approximations. The authors consider that with the use of direct assessments, more reliable prevalence rates would be obtained, probably detecting more cases. The authors predict that direct evaluation and the use of DSM-5 criteria would increase the prevalence of NDDs.

In Spain, studies on the prevalence of NDDs are scarce, despite their importance for establishing a health system based on holistic prevention and targeting from the foundations of the problem, with a cyclical approach that looks beyond a single cause–effect relationship and considers all the circumstances that accompany the clinical manifestations. Assessing the context is as important as—or even more important than—assessing the symptoms themselves.

It is important to recognize certain distinctions, such as clinical populations vs. the general population, rural vs. urban settings, and different levels of socioeconomic resources.

In our review of NDD prevalence studies, we noted that multimorbidity was the norm, as determined by Kita [11] in Japan, Bitta (2018) [15] in low-resource countries, Fleming (2020) [18] in Scotland, Carballal (2017) [20] in Spain and Hansen (2018) in Norway [19].

We also observed that the prevalence remained stable over time in different cultures, ages, ethnicities (Faraone et al., 2020) [24], socioeconomic strata, community types (rural or urban) and religions [17].

Likewise, we found that the differences in sex were consistent, with males being more affected by general psychiatric psychopathology, as reflected in the contributions of Fleming [18] and Dalsgaard [27]. With respect to the studied NDDs and their comorbidities, 66.3% of children included in Hansen's study [19] were male, and Saito [26] reported a male:female ratio of 2.2:1. With respect to ADHD, male:female ratios of 4:1 and 2:1 have been determined (Catalá-López, 2012) [22], generally coinciding with the ratios reported (3–2:1) in the studies by Sayal [23] and Faraone [24]. Finally, in children with ASD, the study by Pérez-Crespo [25] reported a male:female ratio of 4.5:1.

Regarding the variability in the global prevalence of NDDs, the prevalence of single NDDs has been found to range from 4.70% in Scotland [18] to 55.5% in Norway [19] to 88.50% in Japan [11]. It is important to note the possible influence of methodological factors, such as the direct evaluation of children in Japan and Norway, as well as the activity of the countries in detection and diagnosis, with NDDs tending to be underdiagnosed in developing countries. In addition, it would be necessary to analyse the lack of impact of our work with the publication of the DSM-5-TR and how this new version could affect the prevalence of NDDs.

The symptomatology of a disorder is partially a reflection of its context—that is, it is dependent on a combination of internal (genetic) and external (environmental) influences. It is a dialogue between the contextual and the biological, between the social and the individual. The combination of these factors necessitates a multifactorial consideration of epidemiological, clinical and molecular findings in complex diagnoses such as NDDs.

Although it is known that epigenetic changes associated with diseases occur throughout life, the labile nature of the epigenetic state during the first stages of development makes this time especially significant and decisive.

Due to the exponential increase in consultations related to neurodevelopmental problems in paediatrics, we consider it pertinent to carry out and promote studies in real-world populations through direct examinations of the children. Early intervention is essential to improve prognosis and early diagnosis.

## Supplementary Information

**Additional file 1.** Initial search.

## Data Availability

We have data and materials accessible through the main author. Correspondence about the manuscript should be addressed to Dr. Lorena Francés-Soriano.

## Abbreviations

NDD: Neurodevelopmental disorder
ID: Intellectual disability
ADHD: Attention-deficit/hyperactivity disorder
ASD: Autism spectrum disorder
SLD: Specific learning disorder (e.g., dyslexia)
CD: Communication disorder
MD: Motor disorder
TS: Tourette’s syndrome
TD: Tic disorder
DCD: Developmental coordination *disorder*
DD: *developmental dyslexia*
DLD: Developmental language disorder
ODD: Oppositional *defiant* disorder
SLI: Specific language impairment
LAMIC: Low- and middle-income countries
CAMHS: Child and Adolescent Mental Health Services
DSM-5: Diagnostic and Statistical Manual of Mental Disorders, 5^th^ Edition
ICD-10: International Statistical Classification of Diseases and Related Health Problems, *10th* Revision
*ICD**-11*: *International Statistical Classification of Diseases and Related Health Problems, 11th* Revision
WHO: World Health Organization
APA: American Psychiatric Association

## Footnotes

1 Tables and graphs will be inserted throughout the text to facilitate an understanding of the data.
