# Supplementary material for: Current state of knowledge on the prevalence of neurodevelopmental disorders in childhood according to the DSM-5: a systematic review in accordance with the PRISMA criteria
Source: Child Adolesc Psychiatry Ment Health. 2022 Mar 31;16:27. doi: 10.1186/s13034-022-00462-1 (PMC8973738; doi:10.1186/s13034-022-00462-1)
Supplement: Supplementary file 1 — Additional file 1. Initial search. [file 13034_2022_462_MOESM1_ESM.docx]

**INITIAL SEARCH**

The initial search was carried out at 2 different points in time:

**First search**

During the months of January, March, April, May and June 2019, prior to the main search, an exploratory search was conducted. The project was developed according to the *patient, intervention, comparison, outcome* (PICO) strategy. In addition to the main search terms explained in another section of this article, this preliminary search included the terms “autism”, “ASD”, “ADHD”, “learning disorders”, “dyslexia”, “speech disorders”, “communication disorders”, “intellectual disability”, “tics” and “Tourette”.

The main search was more extensive and included a wider range of bibliographic sources (not only articles but also books, theses and institutional reports were reviewed). Furthermore, in addition to the prevalence, risk factors and/or susceptibility to NDDs were included; the educational, correctional and forensic fields were covered. Attention was focused mainly on literature in the English language, although sources in the Spanish language were not rejected. Two investigators participated in the first review: the main researcher and a researcher specializing in SLD. Subsequently, this entire process was reviewed by two experts in the area of ​​child psychiatry and an expert in statistical methodology and methodological designs.

In this first stage of immersion to establish the current state of knowledge, the researchers expanded the time range of the search (covering the last 30 years) and the types of literature considered (various fields). During the first search, the PRISMA criteria were not enforced, which would imply a possible selection bias. We believe it is pertinent to mention this previous search in this section, since one study is included in the definitive review.

We must note that this first search yielded a large amount of information, of which the majority were duplicate articles or too specific and were therefore discarded. This phase has provided us with a total of 84 bibliographic references, which have provided us with a broad view of the subject we wish to address and allowed us to verify that no systematic reviews have been carried out to establish the prevalence of NDDs thus far.

**Second search**

This second search initially focused on the databases PubMed, ScienceDirect, Scopus and ProQuest during June, July, August and September 2021. Three researchers were involved throughout the process.

This search yielded 774 articles were obtained; therefore, it was decided to limit the time interval to the last 10 years in the PubMed and Scopus databases. As for ScienceDirect and ProQuest, it was decided to limit the time interval to the last 12 months due to the large amount of information obtained from these two databases (a total of 5705 articles before the restriction was applied).

In the results collected from Scopus, no new information was extracted, and no studies were identified that were not in any of the databases; therefore, it was decided not to include this database in the final review.

For the search, the terms "neurodevelopmental disorders", "prevalence", "incidence", "childhood" and "diagnosis" were combined using the basic Boolean operator AND.

All search terms and combinations were compiled into a table; each term had previously been entered in MeSH and DeCs Database searches to confirm their validity, evidencing the extensive information available in this area. (See table of searches by terms.)

**Systematic search**

A systematic search was conducted again in July and August 2021. As mentioned, the previous review that had been conducted during 2019 was updated for the design of a subsequent epidemiological study (currently in progress).

The same procedure used in the previous phases was followed again in this phase, but the search was modified with respect to its restrictions. Specifically, the following combination of search terms was used: ((((neurodevelopmental disorders) AND (prevalence)) AND (incidence)) AND (childhood)) AND (diagnosis). From this search, 93 articles were obtained from PubMed, 14 from Scopus, 181 from ScienceDirect and 493 from ProQuest, with the final search performed on August 17, 2021. Based on the titles, 46 articles from PubMed, 10 from Scopus, 160 from ScienceDirect and 481 from ProQuest were discarded. Thus, the final set of articles comprised 4 from Scopus, 45 from PubMed, 24 from ScienceDirect and 8 from ProQuest.

Due to the high volume of articles obtained from ProQuest (n = 493), it was decided to apply an additional filter to the ProQuest search to retrieve only articles that contained all the search terms in the “abstract” section. In addition, the search was limited by type of journal and by specialty; once all restrictions were applied, there were 12 remaining articles to review.

Specifically, the inclusion and exclusion criteria for study selection were as follows:

Inclusion criteria

- Empirical research. Experimental or quasi-experimental designs, systematic reviews, meta-analyses, case studies and randomized studies.

- Published between 2011 and 2021 (for PubMed and Scopus).

- Published between 2020 and 2021 (for ScienceDirect and ProQuest).

- Studies involving a diagnostic approach to ADHD, ASD, language disorder, phonological disorder, motor disorder, tic disorders, Tourette’s syndrome (TS), intellectual disability, borderline IQ and specific learning disorder (including dyslexia and dyscalculia).

- Studies that included a child and adolescent population up to 18 years of age.

- Studies with subjects of both sexes.

Exclusion criteria

- Book chapters, manuals, brochures, surveys, newspaper articles and other types of literature.

- Scientific literature that was not published between 2011 and 2021.

- Studies that did not consider serious mental disorders (such as schizophrenia, bipolar disorder, or substance use disorder) or neurological disorders (epilepsy, cerebral palsy, sleep disorders, etc.).

- Studies of populations over 18 years of age.

- Studies that included any type of pharmacological treatment or intervention programme.

- Studies focusing mainly on possible risk factors.

**Final selection criteria: reading of abstracts**

The abstracts of the articles were read; any article that was deemed ineligible based on its abstract was discarded. The results from the four databases were as follows:

- In Scopus, there were 4 eligible articles that were duplicated in other databases.

- In PubMed, we selected 9 articles, 2 of which were duplicates of included studies, leaving us with 7 eligible articles.

- In ScienceDirect, we identified 3 eligible articles.

- In ProQuest, 2 eligible articles remained once the inclusion and exclusion criteria had been applied.

The articles were discarded mainly if they (1) deviated from the specific search topic; (2) were based on the same datasets as other included studies; or (3) were duplicates of other included studies.

**Manual search**

We included an article from the year 2019 for an overview of the epidemiological situation in ​​Spain (Carballal Mariño et al., 2018), since no study in the Spanish population to date has measured the prevalence rates of mental disorders in childhood and adolescence while meeting all the selection criteria for this review.

A manual search identified 5 new eligible articles, which were added to the 12 selected in the systematic search.

An additional search was carried out in the Wiley Online Library, where we found 3 additional studies that were eligible for the review: Hansen et al. (2018), Shriberg et al. (2019), and Kita et al. (2020). In addition, a direct and specific search of PubMed yielded 2 more studies: Bosch et al. (2021) and Fortes et al. (2015).

Thus, the definitive systematic review ultimately included 17 articles published between 2011-2021, mostly in English, with 15 of the 17 articles published between 2017 and 2020.

**List of abbreviations**

NDD: neurodevelopmental disorder

ID: intellectual disability

ADHD: attention-deficit/hyperactivity disorder

ASD: autism spectrum disorder

SLD: specific learning disorder (e.g., dyslexia)

CD: communication disorder

MD: motor disorder

TS: Tourette’s syndrome

TD: tic disorder

DCD: developmental coordination disorder

DD: developmental dyslexia

DLD: developmental language disorder

ODD: oppositional defiant disorder

SLI: specific language impairment

LAMIC: low- and middle-income countries

CAMHS: Child and Adolescent Mental Health Services

DSM-5: Diagnostic and Statistical Manual of Mental Disorders, 5^th^ Edition

ICD-10: International Statistical Classification of Diseases and Related Health Problems, 10th Revision

ICD*-*11: International Statistical Classification of Diseases and Related Health Problems, 11th Revision

WHO: World Health Organization

APA: American Psychiatric Association

PICO: patient, intervention, comparison, outcome.
